# Circumventing Embryonic Lethality with *Lcmt1* Deficiency: Generation of Hypomorphic *Lcmt1* Mice with Reduced Protein Phosphatase 2A Methyltransferase Expression and Defects in Insulin Signaling

**DOI:** 10.1371/journal.pone.0065967

**Published:** 2013-06-20

**Authors:** Kennen B. MacKay, Yiping Tu, Stephen G. Young, Steven G. Clarke

**Affiliations:** 1 Department of Chemistry and Biochemistry, University of California Los Angeles, Los Angeles, California, United States of America; 2 Molecular Biology Institute, University of California Los Angeles, Los Angeles, California, United States of America; 3 Department of Medicine, University of California Los Angeles, Los Angeles, California, United States of America; 4 Department of Human Genetics, University of California Los Angeles, Los Angeles, California, United States of America; University of Iowa, United States of America

## Abstract

Protein phosphatase 2A (PP2A), the major serine/threonine phosphatase in eukaryotic cells, is a heterotrimeric protein composed of structural, catalytic, and targeting subunits. PP2A assembly is governed by a variety of mechanisms, one of which is carboxyl-terminal methylation of the catalytic subunit by the leucine carboxyl methyltransferase LCMT1. PP2A is nearly stoichiometrically methylated in the cytosol, and although some PP2A targeting subunits bind independently of methylation, this modification is required for the binding of others. To examine the role of this methylation reaction in mammalian tissues, we generated a mouse harboring a gene-trap cassette within intron 1 of *Lcmt1*. Due to splicing around the insertion, *Lcmt1* transcript and LCMT1 protein levels were reduced but not eliminated. LCMT1 activity and methylation of PP2A were reduced in a coordinate fashion, suggesting that LCMT1 is the only PP2A methyltransferase. These mice exhibited an insulin-resistance phenotype, indicating a role for this methyltransferase in signaling in insulin-sensitive tissues. Tissues from these animals will be vital for the *in vivo* identification of methylation-sensitive substrates of PP2A and how they respond to differing physiological conditions.

## Introduction

Tightly controlled protein phosphorylation and dephosphorylation is vital to effective cellular function in mammalian cells [1]. The extent of phosphorylation at a given site is balanced by the opposing actions of protein kinases and protein phosphatases [2]. Over one-third of the murine and human proteome is believed to be phosphorylated by the 540 murine (518 human) protein kinases at tyrosine, serine, and threonine residues [3], [4], [5]. These phosphorylation events overwhelmingly occur on serines and threonines with a ratio of approximately 1800∶200∶1 for pS∶pT∶pY modifications, respectively [6]. Tyrosine phosphorylation, catalyzed by protein tyrosine kinases (coded for by 90 genes in the human genome) is opposed by four families of protein tyrosine phosphatases (encoded by 107 distinct genes, with each phosphatase largely specific for a single substrate) [7], [8]. In contrast to the biological strategy evolved for the removal of tyrosine phosphorylation, the catalysis of phosphate removal from the thousands of substrates of the 428 human protein serine/threonine kinases is catalyzed by only ten protein serine/threonine phosphatases [9]. Unlike a typical tyrosine phosphatase, the major serine/threonine protein phosphatase (PP2A) recognizes a wide variety of substrates involved in many signaling cascades and cellular functions [10].

In order to control such a variety of cellular mechanisms, PP2A is regulated through multiple control points: posttranslational modifications, small molecule activators and inhibitors, the presence of regulatory subunits, as well as other types of protein–protein interactions [10]. A basic level of substrate control is provided by the composition of the heterotrimeric subunits composing PP2A [11]. PP2A exists in the cytosol primarily as a heterodimer consisting of a catalytic (C) and a scaffolding (A) subunit, to which a variety of targeting or regulatory (B) subunits associate, altering the specificity of the phosphatase [12], [13]. The complexity of PP2A composition arises from the enormity of distinct PP2A holoenzyme assemblies formed by proteins coded by the two similar yet non-redundant genes responsible for the catalytic subunit [14], [15], the two non-redundant genes coding for the scaffolding subunit [16], [17], and the four unrelated families of regulatory subunits encoded by multiple genes with a variety of splice variants giving rise to at least 23 different B subunits [10], [11], [18], [19], [20]. The vast array of subunits available for integration into PP2A holoenzymes are themselves regulated by spatial and temporal means as well as a strict regimen of posttranslational modifications [10], [21].

Three covalent modifications of particular interest occur on the C-terminal “tail” of the catalytic subunit, a 6 residue sequence (TPDYFL) which is unstructured and yet highly conserved [10], [13], [22], [23], [24]. Phosphorylation of residue T304 and Y307 has been associated with PP2A deactivation [25], [26], [27], [28], [29], while methylation of the C-terminal carboxyl moiety of L309 alters the assembly and activation of PP2A [30], [31], [32]. Moreover, genetic experiments have revealed that a mutant PP2A with a phosphomimetic residue replacing Y307 is unable to be methylated at L309, yet the same mutations at T304 allows methylation, suggesting likely interplay between these modifications [33], [34]. Charge neutralization may play an important role in the effects of C-terminal methylation on PP2A structure and function [13], [23], [24], [35].

Carboxyl methylation of the C-terminus at L309 is a dynamic process catalyzed by the leucine carboxyl methyltransferase LCMT1 [33], [36], [37] and the predominantly nuclear methylesterase PME-1 [38], [39]. LCMT1 is a class 1 *S*-adenosylmethionine-dependent methyltransferase with PP2A as its only known substrate [12], [13]. Although a knockout of *Lcmt1* in mice was found to be lethal during embryonic development, hindering its study [40], site-specific mutational analysis of the PP2A catalytic subunit has revealed that L309 methylation is necessary for binding of the Bα (PR55) subunit [41], and positively influences the binding of the B′ family members [42], subunits thought to protect against oncogenic transformation [43]. On the other hand, methylation appears to decrease the binding of polyoma middle T and α4 binding [33], [44], [45], [46]. Other B subunit families appear to bind irrespective of C-terminal methylation [34], [47], [48].

The complexity of the interaction between LCMT1 and PP2A has been demonstrated in several previous studies. Neither PP2A loss-of-function mutants, in which active site residues are mutated [33], nor wild-type PP2A that has been subjected to small molecule or protein inhibitors [49], [50], can be methylated by LCMT1. Additionally, peptides mimicking the C-terminal tail of PP2A are not substrates of LCMT1 [37]. Recently, co-crystal structures of LCMT1 with the catalytic subunit of PP2Ac shed light on this complex protein interaction, revealing not only surface interactions between the active site of the methyltransferase and the C-terminal tail of PP2A, but also an interaction between a domain of LCMT1 and the active site of PP2A [13]. These studies suggest an additional role for the methyltransferase in minimizing the activity of free PP2A catalytic subunit by selectively enhancing methylation of the activated PP2A and conversion into appropriate trimeric holoenzymes [13].

In an attempt to elucidate the role of LCMT1 and PP2A methylation in higher organisms, we report the generation of an *Lcmt1* hypomorphic mouse model, detailing the biochemical as well as the phenotypic effects of reduced LCMT1 activity. Although disrupting *Lcmt1* was previously found to be lethal in an *Lcmt1* gene-trap mouse [40], in this report we demonstrate partial LCMT1 activity in mice with a distinct *Lcmt1* gene trap mutation. In these mice, splicing around the insertional mutation leads to the production of intact Lcmt1 transcripts and allows homozygous mice to survive embryonic development. We show that *Lcmt1* expression is affected in a tissue-dependent manner, with the largest decreases observed in cardiac and skeletal muscle, and smaller decreases in brain, liver, and kidney. Decreases in PP2A methylation as well as concomitant increases in demethylation were observed in the *Lcmt1* hypomorphic mice, along with decreases in glucose tolerance and increases in glucose-stimulated insulin secretion.

## Methods

### Ethics Statement

This study was performed in accordance with animal use protocols approved by the University of California at Los Angeles Chancellor's Animal Research Committee (Protocol 1993-109-63).

### Animal Husbandry

Mice were kept on a 12-h light/dark cycle and allowed *ad libitum* access to water and NIH-31 7013 chow (18% protein, 6% fat, 5% fiber, Harlan Teklad, Madison, WI). Mice were housed in same-sex cages with two or three other mice. Breeding animals were housed with one partner. Animals were genotyped at 18 days of age and weaned at 21 days of age.

### *Lcmt1* ^−/−^ Mice

*Lcmt1^+/−^* mice were generated with a BayGenomics mutant embryonic stem cell line CSC099 containing a gene-trap insertion in intron 1 of *Lcmt1* [51]. Mice used in this study were backcrossed at least three times to C57BL/6 mice.

### Genotyping

Tail tip biopsies were used for genotyping; DNA was prepared with Allele-In-One Mouse Tail Direct Lysis Buffer (Allele Biotechnology, San Diego, CA) according to the manufacturer's instructions. The site of gene-trap cassette insertion was identified with PCR reactions of intron 1 of *Lcmt1*. Primers were subsequently designed flanking this site of insertion (forward: CCTTTCTGGGTGAGCTCTTG, reverse: AGATGAGCATCGGAATCTGG; 1899 nucleotide product) as well as primers within the gene-trap cassette itself (forward: ATTATTTGCCCGATGTACGC, reverse: ACATCCAGAGGCACTTCACC; 524 nucleotide product) to genotype these animals. The PCR program used for genotyping consisted of an initial denaturation step of 95°C for 5 min, followed by repeated denaturation at 95°C for 1 min, primer annealing at 61°C for 1 min, and elongation at 72°C for 3 min for 35 cycles, followed by a final elongation step at 72°C for 10 min.

### Southern Blotting

Southern blotting was performed by the UC-Irvine Transgenic Mouse Facility. Briefly, a mouse tail biopsy was collected and DNA was extracted utilizing phenol chloroform extraction. Genomic DNA was digested with the AflII restriction enzyme which does not cut within the gene trap cassette and should yield a 9,812 base pair product containing the gene trap insert and adjacent genomic DNA. After agarose gel electrophoresis, DNA fragments were transferred to a membrane and hybridized with a ^32^P-labeled probe recognizing the β-Geo insert. The probe sequence was as follows: gggcgcccggttctttttgtcaagaccgacctgtccggtgccctgaatgaactgcaggacgaggcagcgcggctatcgtggctggccacgacgggcgttccttgcgcagctgtgctcgacgttgtcactgaagcgggaagggactggctgctattgggcgaagtgccggggcaggatctcctgtcatctcaccttgctcctgccgagaaagtatccatcatggctgatgcaatgcggcggctgcatacgcttgatccggctacctgcccattcgaccaccaagcgaaacatcgcatcgagcgagcacgtactcggatggaagccggtcttgtcgatcaggatgatctggacgaagagcatcaggggctcgcgccagccgaactgttcgccaggctcaaggcgcgcatgcccgacggcgatgatctcgtcgtgacccatggcgatgcctgcttgccgaatatcatggtggaaaatggccgcttttctggattcatcgactgtggccggctgggtgtg.

### RNA Isolation, cDNA Generation, and qPCR

All instruments and bench space were cleaned using RNase Away (Fischer, Torrance, CA). Mice were fasted overnight and sacrificed by carbon dioxide asphyxiation. Tissues were dissected and immediately frozen in liquid nitrogen. Approximately 0.1 g of each tissue was homogenized on ice in 1 ml of tri-reagent (Molecular Research Center, Cincinnati, OH), a commercially available guanidinium thiocyanate-phenol-chloroform mixture [52], using a Polytron homogenizer equipped with a PTA-7 generator. Samples were pulsed seven times for 30 s each with 1 min between pulses to prevent heating of the sample. Samples were subsequently centrifuged at 20,000× *g* for 10 min and the floating layer and pellet removed. A volume of chloroform equivalent to 1/5 of the volume of tri-reagent used was subsequently added to the supernatant, shaken, and further centrifuged at 20,000× *g*. The aqueous phase was saved and added to an equivalent volume of isopropanol. The sample was then centrifuged again for 7 min at 20,000× *g*, decanted, further washed with 70% ethanol, and centrifuged again for 5 min at 20,000× *g*. After decanting the ethanol, the RNA pellet was dried and resuspended in 20 µl of RNase-free water (Qiagen, Valencia, CA). Samples were checked for purity by insuring the absorbance ratio at 260 nm/280 nm was at least 1.9, and for degradation by the presence of intact 18S and 28S rRNA after separation on a 1% agarose gel and staining with ethidium bromide. The RNA pellet was treated with a TURBO DNA-free Kit (Applied Biosystems, Carlsbad, CA) to remove contaminating DNA. Total cDNA was generated using a RETROscript kit (Applied Biosystems). cDNA was plated in a 384-well plate with primers within *Lcmt1* exons 1 and 2 (forward: ACTTGGACGACGAGGGAGT; reverse: GATGCTCGATGTACGGATCA), as well as primers on *Lcmt1* exons 2 and 3 (forward: ATGATCCGTACTCGAGCATC; reverse: CTGACTGACACCATGAACTCG), as well as primers on the *B2m* gene (forward: ATGATCCGTACTCGAGTGGTGCTTGTCTCACTGACC; reverse: TATGTTCGGCTTCCCATTCT). The qPCR experiment was performed on a 7900 HT instrument (Applied Biosystems) and data was analyzed using the 2^−ΔCt^ method [53].

### Western Blotting

Mice were fasted overnight and euthanized in a CO_2_ chamber. Tissues were dissected, weighed, and to each gram of tissue 3 ml of sucrose buffer was added (250 mM sucrose, 10 mM Tris base, 1 mM EDTA, adjusted to pH 7.4 with HCl, with phosphatase (HALT, Thermo-Pierce, Rockford, IL) and protease inhibitors (Complete, Roche, Mannheim, Germany)). Homogenization was performed using a Polytron homogenizer with a PTA-7 generator using seven pulses of 30 s with 1 min on ice between pulses. Extracts were then centrifuged at 20,000× *g* and the supernatants were stored at −80°C. Protein in an aliquot of these soluble extracts was precipitated with trichloroacetic acid and quantified by the Lowry method [54]. Aliquots containing 20 µg of protein were added to 10 µl of a 2× SDS-sample loading buffer (100 mM Tris-HCl, pH 6.8, 200 mM β-mercaptoethanol, 4% SDS, 0.1% bromophenol blue, 20% glycerol) and then brought to a final volume of 20 µl with water and heated for 5 min at 100°C. Unless otherwise indicated, the samples were then loaded into 12-well, 10 cm×10 cm, 4–12% RunBlue SDS gels (Expedeon, San Diego, CA) in an Invitrogen XCell SureLock Mini-Cell apparatus along with rainbow molecular weight markers (RPN-800V, GE Healthcare, Buckinghamshire, England). Electrophoresis was performed at 180 V for 1 h. Proteins were transferred from gels to PVDF membranes (Amersham Hybond-P, GE Healthcare) by electrophoresis at 25 V for 3 h using the Invitrogen Blot Module and NuPAGE transfer buffer (Invitrogen, Grand Island, NY). Membranes were blocked overnight with 5% bovine serum albumin and then incubated with primary antibodies diluted in TBS-T buffer (50 mM Tris, 150 mM NaCl, 0.05% Tween 20, pH 7.4) as described in Table 1. After the blot was washed in TBS-T buffer, it was incubated with dilutions of horseradish peroxidase–labeled secondary antibodies as described in Table 1. Peroxidase activity was visualized after treating the blot with ECL Prime Chemiluminescent Agent (GE Healthcare) and detected on Hyblot CL film (Denville, Metuchen, NJ). Every attempt was made to capture exposures within the linear film response. Film densitometry was performed using ImageJ densitometry software.

**Table 1:** Source of antibodies and immunoblotting protocols.

Target	Name	Source	Dilution	Incubation time	Temperature	Polypeptide size
**deMe-PP2A**	α-PP2A, clone 1D6, 05-421	Millipore	1∶10,000	1 h	25°C	36 kDa
**LCMT1**	α-LCMT1 (4A4) (ab77754)	Abcam	1∶10,000	1 h	25°C	38 kDa
**GAPDH**	α-GAPDH (14C10)	Cell Signaling	1∶40,000	1 h	25°C	37 kDa
**beta-actin**	α-beta actin prepared in rabbit (non-commercial)	Gift from Dr. Emil Reisler	1∶40,000	1 h	25°C	45 kDa
**mouse IgG**	α-mouse IgG prepared in rabbit, HRP secondary #7076	Cell Signaling	1∶100,000	1 h	25°C	NA
**rabbit IgG**	α-rabbit IgG prepared in goat HRP conjugated secondary (ab721))	Abcam	1∶100,000	1 h	25°C	NA

### *In Vitro* PP2A Methylation Assay

LCMT1 activity in soluble mouse brain extracts was determined by taking advantage of the base-lability of methyl esters in a vapor diffusion assay similar to that described previously [55]. Briefly, tissues were collected, homogenized in a sucrose buffer, and processed as described for Western blot analysis above. Protein concentration was determined by the Lowry method after protein precipitation with trichloroacetic acid [54]. The *in vitro* methylation assay was performed by adding 2.5 µl of *S*-adenosyl-l-[*methyl*-^3^H]methionine (1 mCi/1.8 ml; 78 Ci/mmol; PerkinElmer) to 100 µg of protein extract in a 100 mM pH 7.4 Tris-HCl buffer in a total volume of 25 µl. This was incubated at 37°C for 1 h. This reaction was quenched by adding 2× sample loading buffer (100 mM Tris-HCl, pH 6.8, 200 mM β-mercaptoethanol, 4% SDS, 0.1% bromophenol blue, 20% glycerol) and subsequent heating to 100°C for 5 min. These extracts were then loaded onto 4–12% RunBlue SDS gels (Expedeon, San Diego, CA) and separated by electrophoresis alongside molecular weight markers. The gel was then stained with Coomassie Brilliant Blue and the region corresponding to proteins of 31–45 kDa was cut into three slices. These slices were added to microfuge tubes containing 100 µl of 2 M NaOH to release the carboxyl methyl esters as methanol. These uncapped tubes were placed in scintillation vials containing 5 ml of scintillation fluid (Safety-Solve, RPI, Mount Prospect, IL) such that the scintillation fluid never directly contacted the base-treated sample. The vials were then tightly capped and incubated overnight to allow the diffusion of the released [^3^H]methanol into the fluor. Samples were counted three times with a liquid scintillation counter. A parallel lane of the molecular weight ladder was cut and counted as a negative control.

### Glucose Tolerance and Glucose-Stimulated Insulin Secretion Assays

Mice were fasted overnight for 15 h by placing them in fresh cages with water but without food. Blood glucose measurements were recorded with an Accucheck Active blood glucose meter and Accucheck Active glucose test strips (Roche, Mannheim, Germany), requiring ∼1–2 µl of blood per measurement. The tail vein of each animal was nicked with a fresh scalpel, and a glucose test strip immediately spotted with blood and analyzed. From the same tail nick, another 40 µL of blood was collected into 50 µl of PBS-EDTA anticoagulation buffer (10 mM sodium phosphate, 5.4 mM disodium EDTA acid (2 mg/ml), 137.9 mM sodium chloride), immediately placed on ice and saved for the insulin assay. Following this initial measurement, animals were orally administered a bolus of 0.5 g/ml d-glucose dissolved in water corresponding to 2 g glucose per kilogram of body weight. Fresh blood was collected for the insulin assay and glucose levels analyzed at 5, 15, 30, and 60 min following the glucose load. The collected blood samples were spun at 4°C at 1,000× g for 10 min to pellet cells and the plasma insulin level was measured in the supernatant using a Rat/Mouse Insulin ELISA kit (Catalog # EZRMI-13K, EMD Millipore, Billerica, MA) according to the manufacturer's instructions.

## Results

### Generation and characterization of a *Lcmt1* hypomorphic mouse model

**Figure 1:**
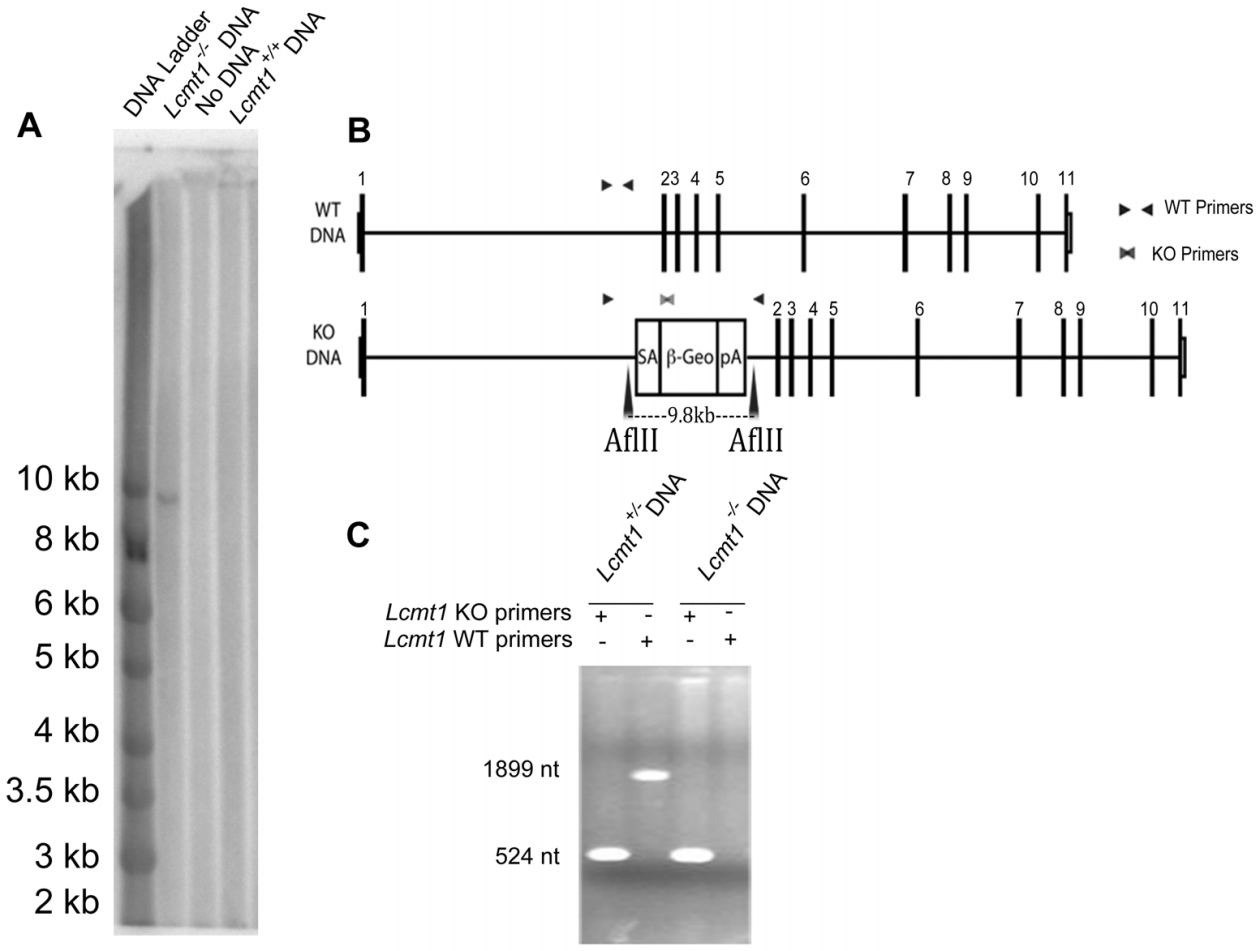
Genomic confirmation of *Lcmt1^−/−^* animals. Panel A: Southern blot showing a single band of 9.8 kDa corresponding to the predicted AflII restriction product. No other bands were seen at other sizes in *Lcmt1^−/−^* gel lane or in the gel lanes of WT DNA samples. Panel B: Schematic of *Lcmt1^+/+^* and *Lcmt1^−/−^* genomic DNA showing the position of the gene-trap insertion, PCR primers, and AflII restriction sites flanking the gene trap cassette. Panel C: Primers flanking the gene-trap cassette insertion site (WT primers) amplify an 1899-bp product when the gene trap is absent; primers inside the gene trap cassette (KO primers) amplify a 524-bp product when the gene trap is present (see “Methods”). When the 8.6-kb gene-trap cassette is present, WT primer amplification is prevented.

High-percentage male chimeric mice were generated with a BayGenomics embryonic stem cell line CSC099 [51] containing an insertional mutation in intron 1 of *Lcmt1* (identified by 5′-RACE). Chimeric mice were bred to create *Lcmt1^+/−^* mice. The precise location of the insertional mutation was determined with a PCR-based screening system using forward primers within intron 1 and a reverse primer on the gene-trap cassette. Sequencing of the PCR products revealed that the gene-trap cassette was located 19,455 nucleotides into intron 1, or at position 19,729 of the gene (counting from the transcriptional start site and the first base of the initiating ATG codon designated as position 168). Sequencing of the insertion site additionally revealed the insertion of 3 exogenous nucleotides at the 5′ end of the insertion site as well as the truncation of 194 nucleotides from the 5′ end of the *β-geo* gene-trap cassette. We confirmed a single non-concatenated gene trap insertion in intron 1 of *Lcmt1* by Southern blotting using a probe within the gene trap cassette (Figure 1A and 1B). This experiment confirmed the presence of the β-geo insert in *Lcmt1* ^−/−^ animals at the predicted site. The absence of multiple bands indicates a single gene trap cassette insertion and the size of the hybridizing band rules out concatemers at the site of insertion. A control with wild-type DNA yielded no signal. Genotyping was accomplished with primers flanking the insertion site (that only amplify in absence of gene trap) and with *β-geo* primers (that only amplify in presence of the gene trap) (Figure 1C).

**Figure 2:**
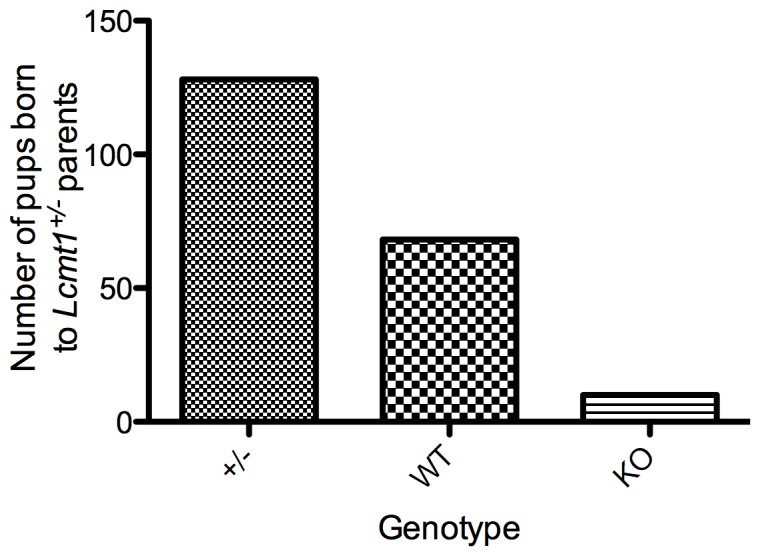
Initial intercrosses of *Lcmt1^+/−^* mice yield lower-than-expected numbers of homozygous offspring. Pups were counted on the day they were born and genotyped at 18 days of age.

F1 heterozygous mice were backcrossed at least three times to C57BL/6 mice. Homozygous mice were initially generated by intercrossing heterozygotes; homozygous offspring were obtained at a lower-than-predicted frequency (Figure 2). In part, the low frequency of homozygous offspring probably was due to the death of *Lcmt1^−/−^* embryos during development as many resorbing embryos were observed at E9.5. Fewer than one pup was lost from birth until the time of weaning at 18 days of age, so it is unlikely that postnatal death accounted for the reduced frequency of *Lcmt1^−/−^* mice. *Lcmt1^−/−^* mice that survived development exhibited normal vitality and were fertile; intercrossing *Lcmt1^−/−^* mice yielded litters of 5–8 pups. *Lcmt1^−/−^* mice were healthy and had a normal lifespan.

**Figure 3:**
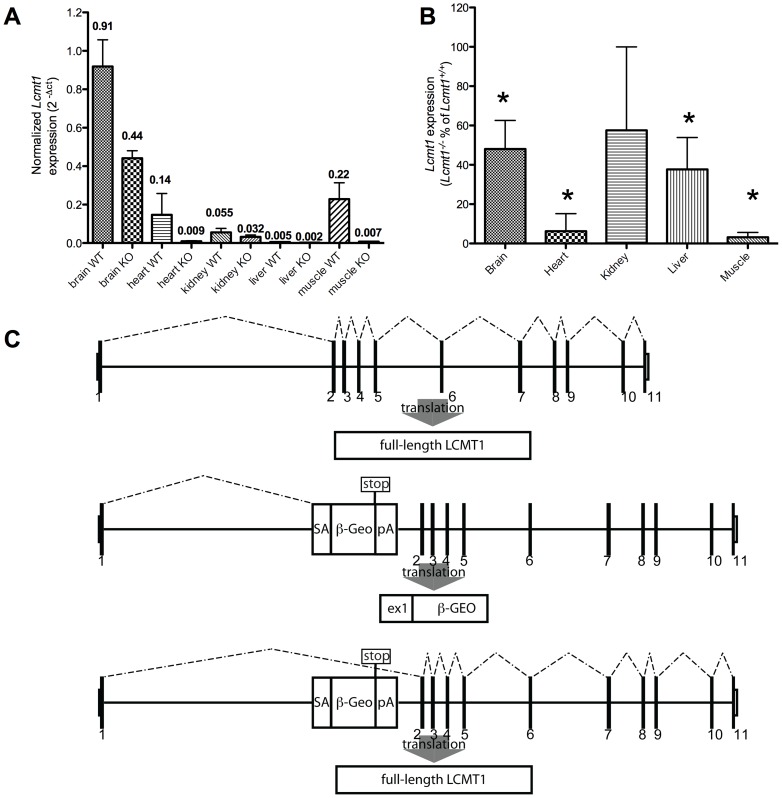
*Lcmt1* transcript quantitation in wild-type and *Lcmt1^−/−^* mouse tissues. Panel A: *Lcmt1* transcript levels from five tissues, measured using primers flanking exons 1 and 2, and exons 2 and 3 as described in the “Methods” section, were normalized to β-2 microglobulin transcript levels measured in the same tissue. ΔCt values are displayed above each column. Values were averaged from tissues prepared from three wild-type and three knockout mice; all qPCR determinations were performed in duplicate. Errors bars show the standard deviation. Panel B: *Lcmt1^−/−^* transcript levels as a percent of *Lcmt1^+/+^* transcript levels. Error bars represent the standard deviation. Asterisks indicate where the decrease in *Lcmt1^−/−^* transcript levels compared to those of wild-type mice are significant at a *p* value of less than 0.05 by the Student's *t*-test. Panel C: the upper schematic shows the generation of full-length *Lcmt1* mRNA in wild-type mice while the middle schematic shows the generation of a truncated transcript in *Lcmt1^−/−^* mice. The lower schematic shows how alternative splicing can skip over the gene trap cassette to produce a full-length mRNA transcript.

The analysis of brain, heart, kidney, liver, and skeletal muscle tissues from *Lcmt1^−/−^* mice revealed lower levels of *Lcmt1* transcripts than in wild-type mice, but not the complete elimination of transcripts that we had expected (Figure 3A). Interestingly, the decrease in *Lcmt1* transcripts appeared to be tissue specific with kidney exhibiting the highest *Lcmt1* expression levels relative to wild-type mice at 58%, followed by brain at 48%, liver at 37%, heart at 6% and skeletal muscle at just 3% (Figure 3B). Analysis of the *Lcmt1* primary genomic sequences as well as that of the insertion site revealed that the splice donor site on exon 1 of *Lcmt1* is a non-consensus site. With the loss of a branch site on the gene trap cassette, this leads to the *in silico* prediction of partial splicing around the gene trap cassette (Figure 3C) [56]. To rule out the presence of alternative transcriptional start sites, we performed qPCR experiments, comparing the production of PCR products using exon 2 and 3 primers with products obtained with exon 1 and 2 primers. Both primer pairs yielded similar transcript levels via qPCR, implying that the *Lcmt1* transcripts were generated by splicing around the gene-trap cassette. Had there been an alterative transcriptional start site 3′ of the insertional mutation, we would have expected to find much higher *Lcmt1* expression levels with the exon 2/3 primer pair. These data indicate that the *Lcmt1^−/−^* mice are homozygotes for a hypomorphic allele rather than for a *bona fide* knockout allele.

**Figure 4:**
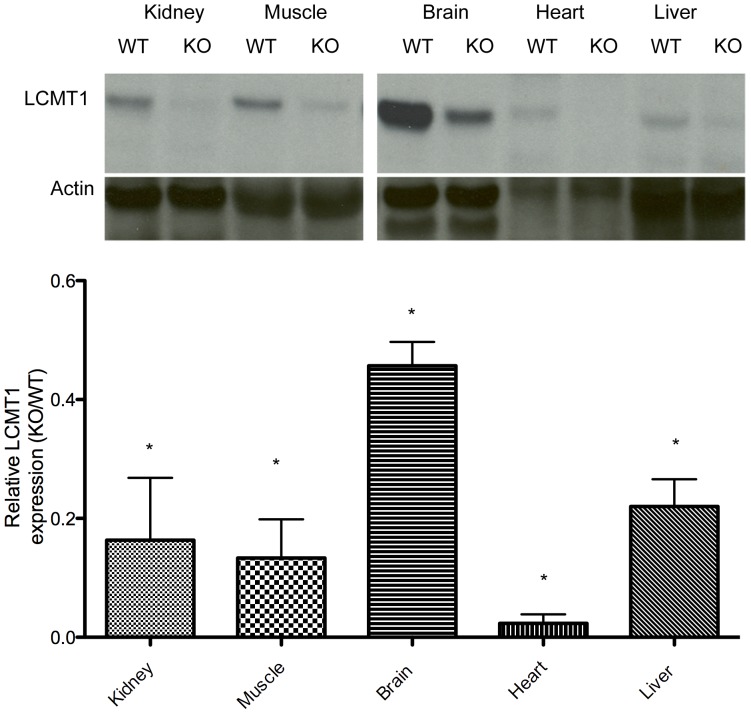
Quantitation of LCMT1 levels in tissues of *Lcmt1^−/−^* animals compared to wild-type controls. Panel A: Representative Western blots of tissue extracts with antibodies against LCMT1. Antibodies to β-actin were used as a loading control. All samples were electrophoresed on the same gel along with protein ladder standards. Panel B: Summary of results from experiments with extracts from three wild-type and three *Lcmt1^−/−^* mice. Densitometry was used to normalize LCMT1 levels to actin. The ratio of *Lcmt1^−/−^* to *Lcmt1^+/+^* signals were then plotted with the error bars indicating the standard deviation of three experiments. In each case, extracts of wild-type and knockout tissues were electrophoresed on the same gel as shown in Panel A. Asterisks indicate significant decreases in expression (*p* value less than 0.05) by Student's *t*-test.

To confirm the presence of a normal-sized LCMT1 protein in *Lcmt1^−/−^* animals, we immunoblotted tissue extracts from *Lcmt1^−/−^* and *Lcmt1^+/+^* mice with antibodies against LCMT1. These experiments revealed that the LCMT1 protein was of normal size but the quantity of the protein was decreased in a tissue-specific fashion similar to that observed with *Lcmt1* transcript levels (Figure 4A; Figure S1). The largest decreases in expression were observed in muscle and heart tissue with *Lcmt1^−/−^* animals having LCMT1 protein levels of only about 13% and under 2% of those in wild-type mice, respectively (Figure 4B). To ascertain the relative levels of LCMT1 in heart tissue in *Lcmt1^−/−^* animals as compared to wild-type controls, we performed additional blotting using 100-fold higher concentrations of the primary LCMT1 antibody and a 24 h primary antibody incubation (Figure S2, Panel A). These experiments showed that LCMT1 levels in *Lcmt1^−/−^* heart tissue represent less than 1% of those of the wild type (Figure S2; Panels B and C). Decreased expression of LCMT1 protein level was also found in kidney, liver, and brain, with levels in knockout tissues compared to wild-type tissues of about 16%, 22%, and 46%, respectively (Figure 4). With the exception of kidney, the relative LCMT1 expression appeared to be consistent with the mRNA levels (Figure 3B). In wild-type mice, LCMT1 protein was most abundant in brain, followed by muscle, kidney, and liver; the lowest expression was observed in heart (Figure 4). These levels correspond to those previously published in a transcriptome study [57].

### *Lcmt1^−/−^* animals display decreased methylation of PP2A

**Figure 5:**
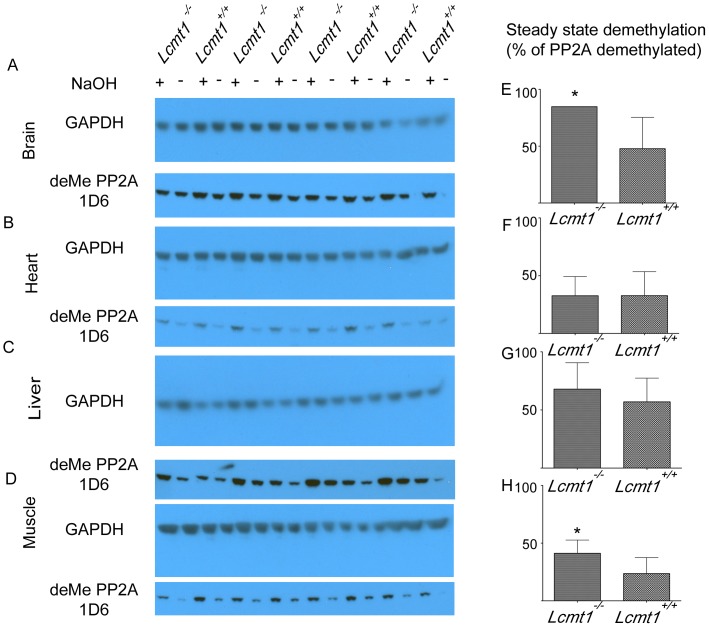
Quantitation of the steady state methylation level of PP2A in tissues of *Lcmt1^−/−^* and *Lcmt1^+/+^* mice. Panel A–D: Tissue extracts from four wild-type and four knockout animals were treated either with 0.1 M NaOH for 1 min at room temperature to cleave methyl esters followed by addition of 0.1 M HCl and 0.5 M Tris-HCl, pH 7.4 to neutralize the solution (NaOH +), or a previously neutralized buffer containing the aforementioned solutions as a control (NaOH −). Polypeptides from these extracts were then separated by SDS-PAGE using 17-well, 4–12% Bis-Tris NuPAGE gels (Invitrogen), transferred to PVDF membranes, and incubated with clone 1D6 antibodies selective for the demethylated catalytic subunit of PP2A, and antibodies to GAPDH as a loading control. These procedures are described in detail in “Methods”. Panels E–H: The percent of the PP2A catalytic subunit that is demethylated was calculated as described by Yu *et al.* [33]. The columns represent the average ± the standard deviation for the four *Lcmt1^−/−^* and four *Lcmt1^+/+^* mice shown in Panel A–D. Statistical significance at the level of *p* less than 0.05 was determined by the Student's *t*-test and is denoted by the asterisk.

Decreased levels of LCMT1 in the *Lcmt1^−/−^* mice did not appear to affect the cellular levels of the catalytic subunit of PP2A but did reduce methylation of PP2A in brain, liver, and skeletal muscle (Figure 5). Analysis of the steady-state level of demethylated PP2A by Western blotting indicated that the decrease in methylation in *Lcmt1^−/−^* animals was statistically significant in brain and skeletal muscle tissue. We detected no difference in the steady state of the demethylated form of PP2A in heart, and a statistically insignificant gain of demethylation in liver. Interestingly, the largest decrease in LCMT1 expression in *Lcmt1^−/−^* animals was in heart although it appeared this did not alter methylation of PP2A in this tissue. *Lcmt1^+/+^* skeletal muscle tissue was found to have an average steady-state methylation level of about 77%, which was found to decrease to an average of about 59% in *Lcmt1^−/−^* animals. In brain, which was found to have steady-state methylation of about 52%, we observed a drop to about 15% in *Lcmt1^−/−^* animals.

**Figure 6:**
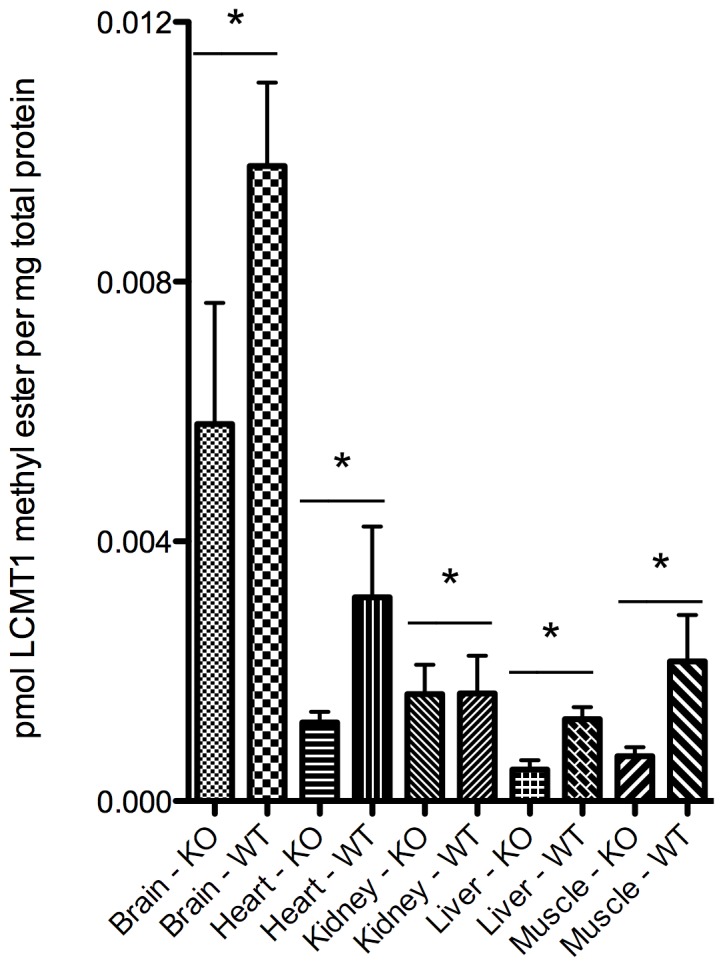
Quantification of *in vitro* PP2A methylation. Extracts from *Lcmt1^−/−^* and *Lcmt1^+/+^* mice were incubated with [^3^H]AdoMet as described in the “Methods.” The radioactive peak of [^3^H]methyl esters corresponding to the position of the PP2A catalytic subunit at ∼36 kDa was quantified after the background (from a parallel lane of molecular weight standards) was subtracted. Each column represents the mean of three independent experiments utilizing tissue extracts from three *Lcmt1^−/−^* and three *Lcmt1^+/+^* mice. Error bars represent the standard deviation. Asterisks indicate statistically-significant differences between extracts from wild-type and knockdown mice (*p* value less than 0.05) with the Student's *t*-test.

An *in vitro* methyltransferase assay utilizing endogenous LCMT1 in cytosolic extracts revealed a similar decrease in PP2A methylation (Figure 6). With the exception of extracts from kidney, LCMT1 activity significantly decreased in *Lcmt1^−/−^* animals with the largest decreases of 62 to 68% in liver, heart, and skeletal muscle. Brain exhibited a 41% reduction in methylation.

### *Lcmt1^−/−^* animals display increased insulin resistance

**Figure 7:**
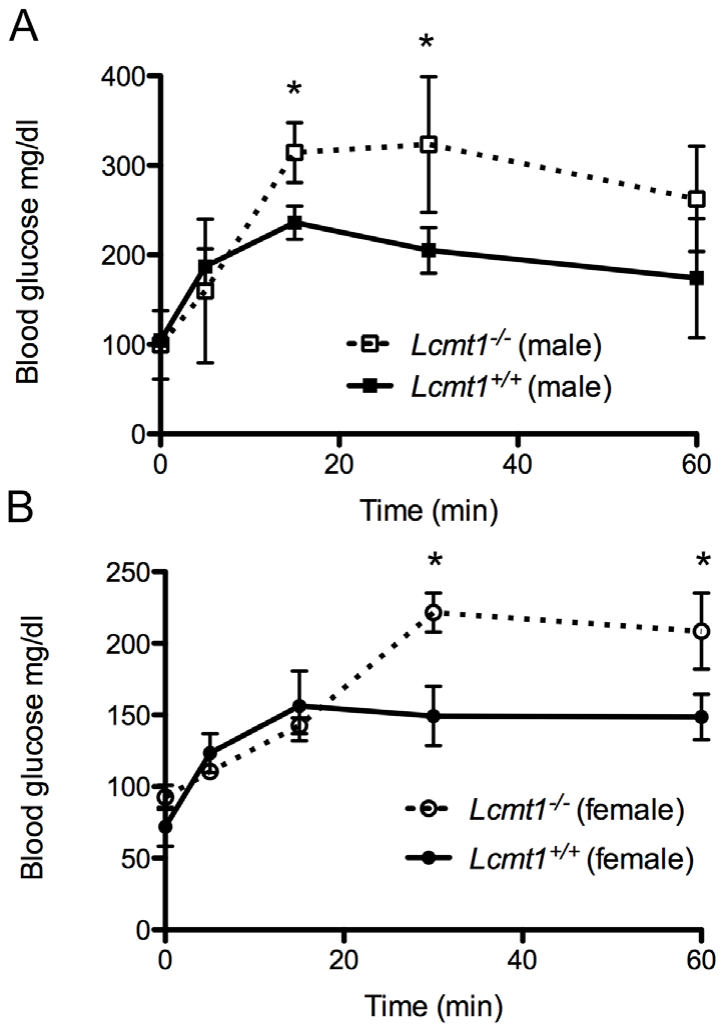
Decreased glucose tolerance in *Lcmt1^−/−^* mice. Male and female mice were fasted overnight and administered 2 g of glucose orally per kg body mass followed by measurement of whole blood glucose levels as described in the “*Methods*.” Panel A: Results from three wild-type and three knockout male mice. Error bars represent standard deviation; asterisks indicate statistically-significant differences between wild-type and knockout mice (*p* value less than 0.05) with the Student's *t*-test. Panel B: Results as in panel A but using 3 wild-type and 3 knockout female mice.

**Figure 8:**
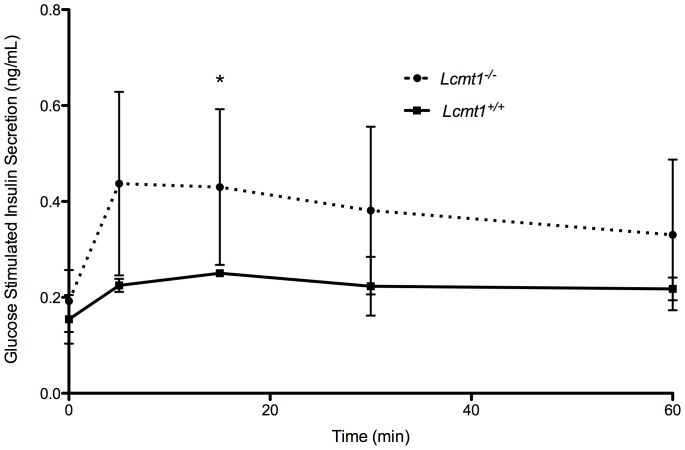
Male *Lcmt1^−/−^* animals display increased glucose-stimulated insulin secretion. Three wild-type and three knockout mice were fasted overnight and administered 2 g of glucose per kg of body mass, followed by measurement of plasma insulin levels as described in the “*Methods*.” Error bars represent standard deviations; asterisks indicate statistically-significant differences between wild-type and knockout mice (p value less than 0.05) with the Student's *t*-test.

Because PP2A is involved in halting kinase cascades involved in growth and cell signaling [58], we next looked at how a decrease in PP2A methylation would affect insulin signaling. *Lcmt1^−/−^* mice were fasted overnight for 15 h and subsequently given an oral bolus of glucose (2 g/kg of body weight). Following the glucose load, blood samples were obtained for blood sugar and insulin measurements. *Lcmt1^−/−^* animals displayed significantly decreased glucose tolerance (Figure 7) despite higher insulin levels (Figure 8), a pattern often associated with insulin resistance [59], [60], [61].

## Discussion

In this study, we present a novel mouse model that is hypomorphic for LCMT1, the carboxyl-terminal protein methyltransferase that methylates PP2A, a major protein phosphatase. We had expected that the gene-trap allele would yield a total knockout, but splicing around the insertional mutation allowed for some expression of *Lcmt1* transcripts. Complete loss of LCMT1 results in embryonic lethality [40], but the hypomorphic Lcmt1 mice survived, making it possible to assess the effect of reduced methylation of the PP2A catalytic subunit. *Lcmt1^−/−^* animals were originally obtained at a lower-than-predicted frequency, but surviving *Lcmt1^−/−^* mice appeared healthy and were fertile.

*Lcmt1^−/−^* mice had reduced levels of *Lcmt1* transcripts as well as decreased LCMT1 protein levels. Additionally, the only known substrate of LCMT1, the protein phosphatase PP2A, displayed reduced steady state methylation levels. Furthermore, *in vitro* methyltransferase assays revealed decreased LCMT1 methyltransferase activity. *In vitro* methyltransferase experiments are complicated by the relationship between the methyltransferase, PP2A, and the presence of the PP2A-specific methylesterase PME-1. To avoid demethylase activity, cellular membranes and the nucleus was removed, making it possible to exclude the predominantly nuclear PME-1. However, our results, as well as previous results [33], [43], suggest PP2A is largely methylated *in vivo*, suggesting *in vitro* analyses utilizing [^3^H]AdoMet as a methyl donor may be limited by the availability of nonmethylated PP2A substrate in tissues.

PP2A has been associated with control of the major serine-threonine kinases in growth and signaling and as such has long been implicated in insulin signaling and the onset of insulin resistance [62]. Several studies have implicated LCMT1 in the direct control of insulin signaling. For example, glucose directly controls PP2A methylation in isolated pancreatic β-cells [63] and depletion of PP2A in pancreatic β-cells attenuates glucose-stimulated insulin secretion [64]. It has also been suggested that inhibition of PP2A is a leading cause of insulin resistance in heart tissue [65], [66]. Our findings indicate that decreased methylation of PP2Ac due to decreased expression of LCMT1 contributes to decreased glucose tolerance and increased glucose-stimulated insulin secretion, lending additional support to the previous findings.

Significant evidence exists implicating a role for PP2A in the onset of Alzheimer's disease and the formation of hyperphosphorylated tau in neurofibrillary tangles [67], [68], [69]. Endogenous peptide inhibitors of PP2A are increased in brains of Alzheimer's disease patients [70], implicating loss of phosphatase function in the disease. Later, it was discovered that PP2A influenced tau phosphorylation both directly by catalyzing tau dephosphorylation and indirectly through regulation of GSK-3β [71], one of the major tau kinases [72]. Of interest to this study, experiments in animal models showed that adding a methyltransferase inhibitor was capable of inducing the tau hyperphosphorylation [73]. Further evidence suggesting a correlation between tau hyperphosphorylation and reduced methylation of PP2A arose from evidence suggesting high plasma homocysteine levels correlated with demethylated PP2A and Alzheimer's disease [74], and that downregulation of LCMT1 correlated with tau hyperphosphorylation [68]. Increased expression of LCMT1 in neuroblastoma cells has also been shown to alter actin assembly, promoting tau-related processes and inducing neuritogenesis [75]. In humans, however, despite evidence suggesting a role for LCMT1 and PP2A in Alzheimer's disease [68], [76], genetic variation of these genes did not appear to alter risk for late-onset Alzheimer's disease [77]. Mice expressing a dominant-negative version of PP2A containing an L309A mutation have been shown to exacerbate tauopathies when backcrossed with neurofibrillary tangle-forming mice containing a P301L mutation in tau [78]. Although we investigated this finding by blotting with antibodies specific to hyperphosphorylated tau in neurofibrillary tangles, we did not observe significant differences between *Lcmt1^−/−^* and *Lcmt1^+/+^* animals (data not shown). However, because the animals utilized in this study were under 100 days of age, changes in *Lcmt1^−/−^* tau brain chemistry could become apparent later in animal life, a possibility that we have not investigated.

Dysregulation of PP2A has been associated with cellular proliferation and oncogene transformation [79]. Specifically, methylation-sensitive subunits are implicated in controlling cell growth and survival [80], [81], [82], [83]. Recently, it has been suggested that viral proteins can induce oncogenic transformation through the replacement of methylation-sensitive PP2A B subunits with methylation-independent viral proteins [84]. This replacement may circumvent the antigrowth and antiproliferative effects of methylation-sensitive PP2A heterotrimers and suggests that reduced LCMT1 activity could contribute to oncogenic transformation and cancer [43]. Our observations of the hypomorphic *Lcmt1^−/−^* mouse model over a period of several years have not provided any evidence for an increased frequency of externally visible tumors. However, we cannot exclude changes in the rate of spontaneous oncogenic transformations and oncogenic growth in the hypomorphic *Lcmt1* mice.

## Supporting Information

Figure S1 **Quantitation of LCMT1 levels in tissues of** ***Lcmt1^−/−^*** **animals compared to wild-type controls.** Polypeptides were separated from mouse tissue extracts and Lcmt1 levels were measured by Western blotting as described in Figure 4. Data are shown from all four of the replicate experiments with multiple exposures to obtain densitometric traces in the linear range that were used in the quantitation in Figure 4.
(TIF)

Figure S2 **Quantitation of LCMT1 levels in heart tissue of** ***Lcmt1^−/−^*** **animals compared to wild-type controls.** Panel A. Four wild-type and four knockout heart lysates were blotted for LCMT1 and GAPDH (loading control). Three exposures are shown to obtain the KO and WT LCMT1 signals in the linear range of the film. Panel B: Quantitation of signals by densitometry using ImageJ software is shown with the y-axis reflecting arbitrary density units. Panel C: the KO LCMT1 density is shown as a percent of WT signal for the 3 independent exposures. Because we did not capture KO and WT signals within the linear range of a single film exposure, we can only conclude that LCMT1 in the heart of knockout animals is present at less than 1% of the level in wild-type animals.
(TIF)
