# Supplementary figures and images for: Circumventing Embryonic Lethality with Lcmt1 Deficiency: Generation of Hypomorphic Lcmt1 Mice with Reduced Protein Phosphatase 2A Methyltransferase Expression and Defects in Insulin Signaling
Source: PLoS One. 2013 Jun 20;8(6):e65967. doi: 10.1371/journal.pone.0065967 (PMC3688711; doi:10.1371/journal.pone.0065967)

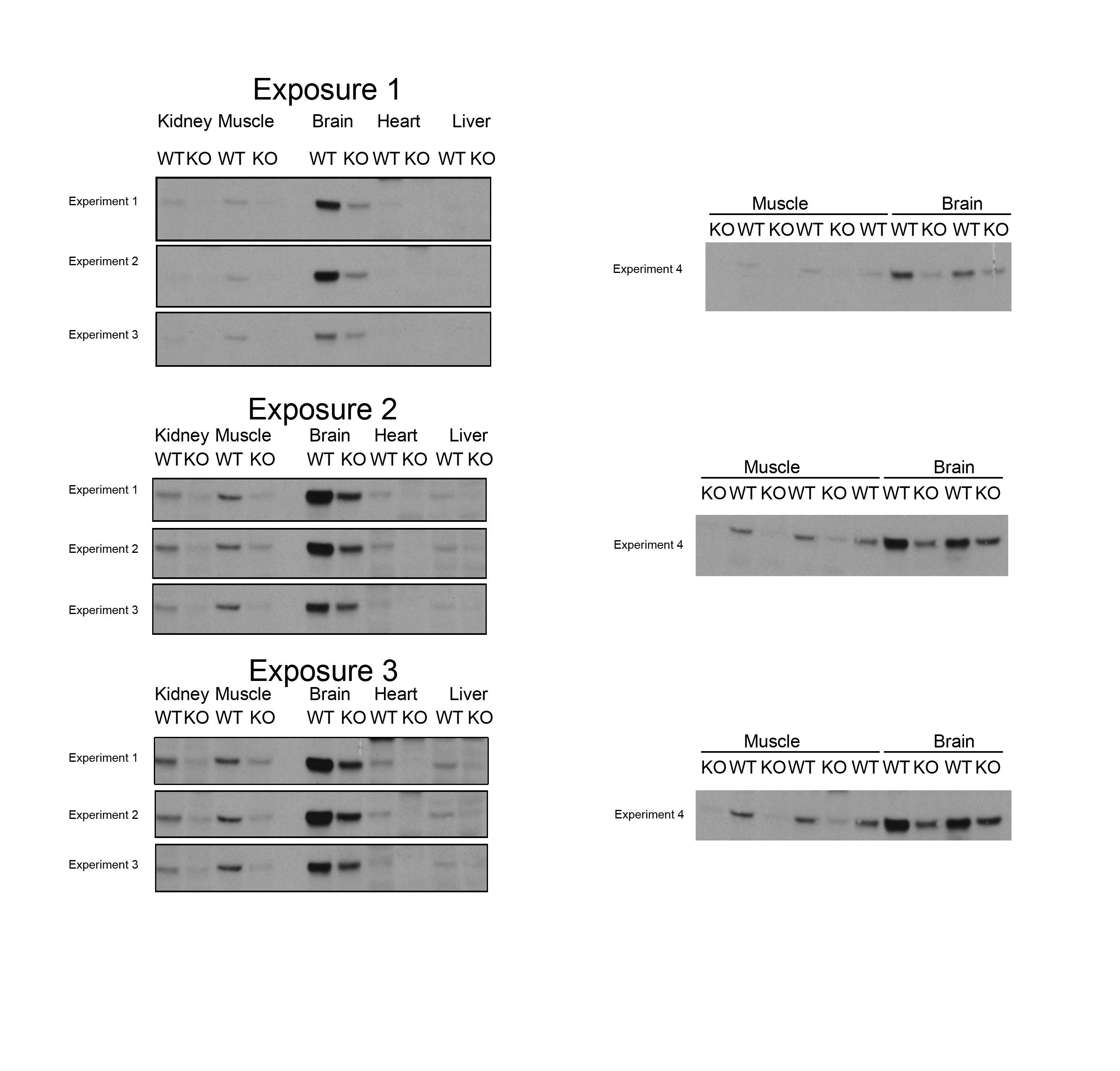

Supplement: Figure S1 — Quantitation of LCMT1 levels in tissues of Lcmt1−/− animals compared to wild-type controls. Polypeptides were separated from mouse tissue extracts and Lcmt1 levels were measured by Western blotting as described in Figure 4. Data are shown from all four of the replicate experiments with multiple exposures to obtain densitometric traces in the linear range that were used in the quantitation in Figure 4. (TIF) [file pone.0065967.s001.tif]

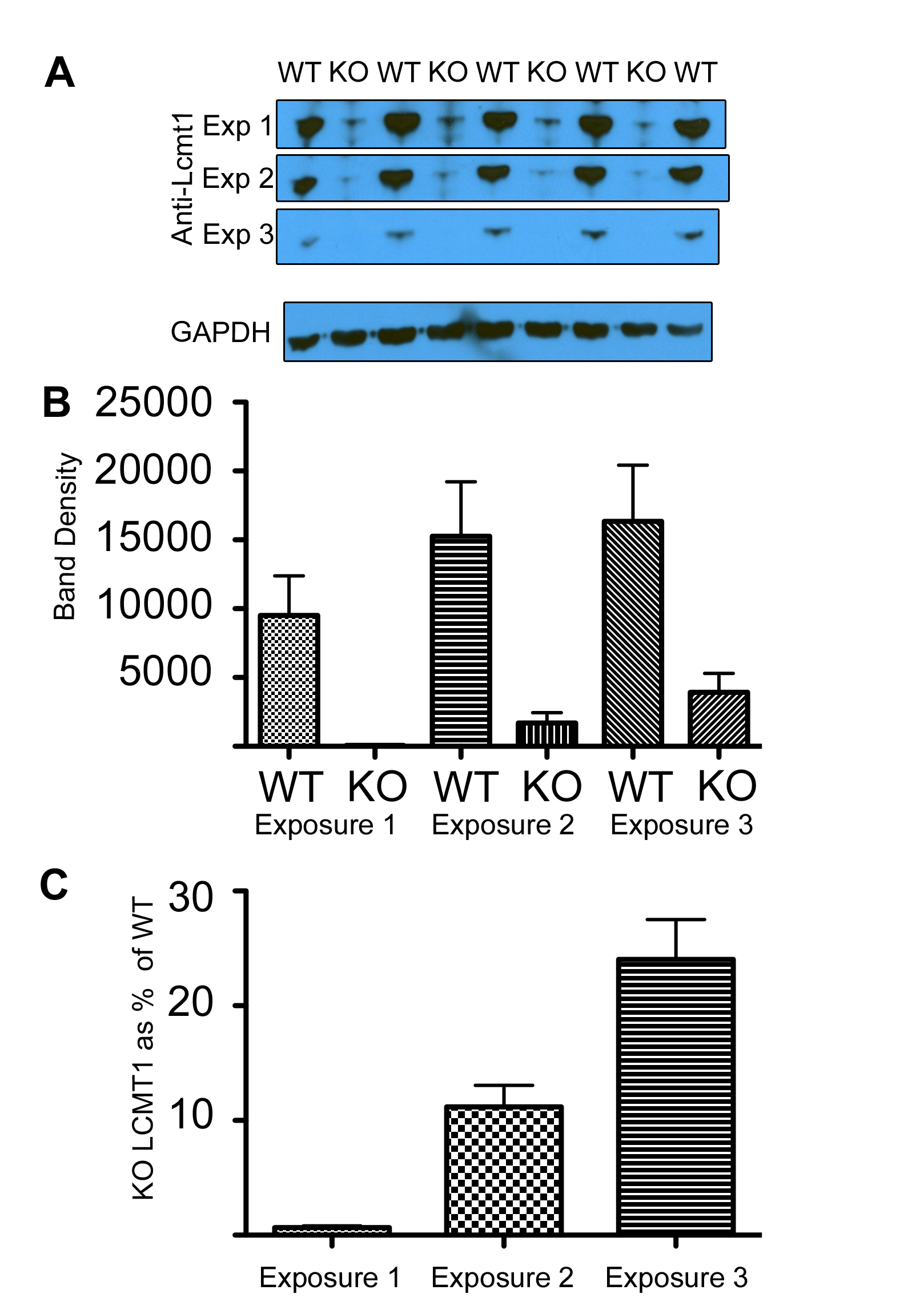

Supplement: Figure S2 — Quantitation of LCMT1 levels in heart tissue of Lcmt1−/− animals compared to wild-type controls. Panel A. Four wild-type and four knockout heart lysates were blotted for LCMT1 and GAPDH (loading control). Three exposures are shown to obtain the KO and WT LCMT1 signals in the linear range of the film. Panel B: Quantitation of signals by densitometry using ImageJ software is shown with the y-axis reflecting arbitrary density units. Panel C: the KO LCMT1 density is shown as a percent of WT signal for the 3 independent exposures. Because we did not capture KO and WT signals within the linear range of a single film exposure, we can only conclude that LCMT1 in the heart of knockout animals is present at less than 1% of the level in wild-type animals. (TIF) [file pone.0065967.s002.tif]
